# A Reliable Criterion for the Correct Delimitation of the Foveal Avascular Zone in Diabetic Patients

**DOI:** 10.3390/jpm13050822

**Published:** 2023-05-12

**Authors:** Guisela Fernández-Espinosa, Carlos Ruiz-Tabuenca, Elvira Orduna-Hospital, Isabel Pinilla, Francisco J. Salgado-Remacha

**Affiliations:** 1 Aragon Institute for Health Research (IIS Aragon), 50009 Zaragoza, Spain; guisela.fernandez3@gmail.com (G.F.-E.); ipinilla@unizar.es (I.P.); 2 Departamento de Física Aplicada, Universidad de Zaragoza, 50009 Zaragoza, Spain; 760104@unizar.es (C.R.-T.); fjsalgado@unizar.es (F.J.S.-R.); 3 Department of Ophthalmology, Lozano Blesa University Hospital, 50009 Zaragoza, Spain; 4 Departamento de Cirugía, Universidad de Zaragoza, 50009 Zaragoza, Spain

**Keywords:** deep capillary plexus, diabetes mellitus, diabetic retinopathy, foveal avascular zone, optical coherence tomography angiography, superficial capillary plexus

## Abstract

Background: Manual segmentation of the Foveal Avascular Zone (FAZ) has a high level of variability. Research into retinas needs coherent segmentation sets with low variability. Methods: Retinal optical coherence tomography angiography (OCTA) images from type-1 diabetes mellitus (DM1), type-2 diabetes mellitus (DM2) and healthy patients were included. Superficial (SCP) and deep (DCP) capillary plexus FAZs were manually segmented by different observers. After comparing the results, a new criterion was established to reduce variability in the segmentations. The FAZ area and acircularity were also studied. Results: The new segmentation criterion produces smaller areas (closer to the real FAZ) with lower variability than the different criteria of the explorers in both plexuses for the three groups. This was particularly noticeable for the DM2 group with damaged retinas. The acircularity values were also slightly reduced with the final criterion in all groups. The FAZ areas with lower values showed slightly higher acircularity values. We also have a consistent and coherent set of segmentations with which to continue our research. Conclusions: Manual segmentations of FAZ are generally carried out with little attention to the consistency of the measurements. A novel criterion for segmenting the FAZ allows segmentations made by different observers to be more similar.

## 1. Introduction

Diabetic retinopathy (DR) is the most severe and frequent ophthalmic complication of diabetes mellitus (DM) and is one of the main causes of blindness worldwide [1]; there is an expected increase in the estimated prevalence of DM, reaching up to 629 million patients worldwide by 2045 [2]. The most frequent DM types are type 1 (DM1) and type 2 (DM2). DM1 appears due to autoimmune β-cell destruction, usually leading to absolute insulin deficiency, whereas DM2 is caused by a progressive loss and dysfunction of β-cell insulin secretion. Both types are heterogeneous diseases due to genetic and environmental factors that result in hyperglycaemia [3].

In clinical practice, the control and diagnosis of DR is mainly based on the assessment of best corrected visual acuity (BCVA), examination of the eye fundus, fluorescein angiography, and evaluation of retinal morphology and thickness using optical coherence tomography (OCT). Nevertheless, patients with no retinal signs of DR can have affected visual function. New technological advances have improved OCT resolution. The introduction of OCT angiography (OCTA) allows a better evaluation of vascular changes by studying retinal vascularization and the foveal avascular zone (FAZ) [4].

OCTA has recently emerged as a powerful noninvasive technique for imaging the microvasculature of the retina based on the evaluation of blood flow imaging [5]. It is performed by acquiring repeated OCT B-scans at the same location to detect changes in the reflectance signal from the movement of red blood cells through blood vessels in volumetric OCT scans. Thus, it allows the visualisation of the superficial (SCP), intermediate (ICP) and deep (DCP) capillary plexuses, the radial peripapillary capillary network, the choriocapillaris (CC) and, partially, the choroidal great vessels and the study of the FAZ [4,6,7].

DR signs can be detected by OCTA, including microaneurysms (MA), capillary nonperfusion areas, intraretinal microvascular abnormalities (IRMA), neovascularisation (NV) and FAZ abnormalities in size and morphology. Vascular density, retinal perfusion and FAZ changes are the most frequently investigated OCTA quantitative parameters correlated with the severity of DR and BCVA. Moreover, OCTA allows the detection of early microvascular changes in DM even before they are detected in the fundoscopy exam [8,9,10,11]. FAZ changes could be observed and measured in SCP and DCP [12,13]. In many OCTA devices, the FAZ needs to be manually delimited. Some of the criteria used to evaluate DR microvascular status are based on FAZ measurements. However, manual segmentations of the FAZ are generally carried out with little care given to measurement consistency. Thus, manually delineating the FAZ results in a large variability, both interobserver and intraobserver, especially in pathological retinas [14]. In addition, the definition of the FAZ is far from being a truly objective criterion. In this paper, we present the problems encountered when manually delimiting the FAZ on the OCTA image, analysing the results obtained by several observers.

The initial point of this study was to show both the interobserver and intraobserver variability of the manual delimitations of the FAZ in patients with DM2 and moderate nonproliferative DR (NPDR) without diabetic macular edema (DME), patients with DM1 without visible DR signs and healthy subjects. Based on the results, we agree on a common new criterion for the delimitation of the FAZ in order to reduce the variability produced by subjectivity in segmentation. In this way, we managed to obtain a really reliable and consistent set of segmentations with which to continue our future research.

## 2. Materials and Methods

### 2.1. Sample Description

We performed a prospective study including a total of 167 eyes. The FAZ from 73 eyes from healthy subjects, 40 eyes from DM1 patients without visible DR signs and 54 eyes from DM2 patients with NPDR without diabetic macular edema DME were studied. The DM2 patients with NPDR were diagnosed during this ophthalmology visit. The study was approved by the “Comité de Ética de la Investigación Clínica de la Comunidad de Aragón” (CEICA) with reference PI19/252 and complies with the principles of Helsinki. All participants were informed, and they signed consent forms.

The exclusion criteria for all participants included amblyopia or BCVA less than 20/40 on the Snellen chart, refractive error over 5.50 diopters (D) of spherical equivalent (SE) or 3.00 D of astigmatism, intraocular pressure (IOP) higher than 20 mmHg, history of any pathology affecting central vision (DME, cataract, age-related macular degeneration, pathologic myopia, macular hole, macular epiretinal membrane, traumatic retinal changes, macrovascular changes, glaucoma with perimetric involvement or papillary atrophy), or inability to perform good-quality OCT and OCTA evaluation (difficulty in layer segmentation, media opacification, or lack of fixation or cooperation).

### 2.2. Experimental Protocol

The participants underwent a complete ophthalmological evaluation including BCVA expressed in logarithms of the minimum resolution angle (logMAR) measured with the Early Treatment Diabetic Retinopathy Study (ETDRS) test, IOP measured by Goldmann tonometry, axial length (AL) measured with an Aladdin KR-1 W Series optical biometry system (Topcon Corporation, Tokyo, Japan) and OCTA.

**Figure 1:**
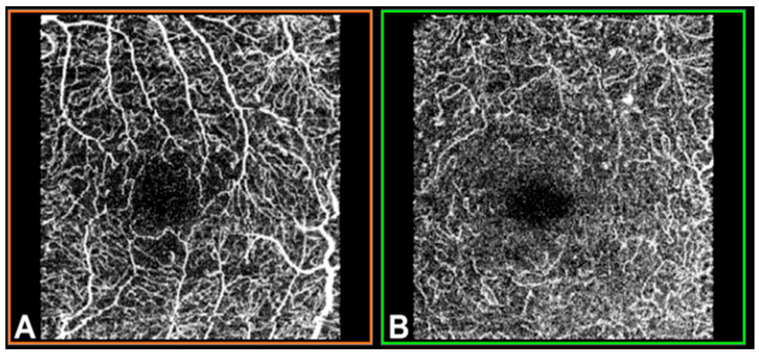
Optical coherence tomography angiography (OCTA) in a 3×3 mm macular area of the left eye, measured by deep-range imaging (DRI) Triton swept-source (SS)-OCT. (**A**) Superficial capillary plexus; (**B**) deep capillary plexus.

OCTA was performed using swept-source (SS)-OCT with deep-range imaging (DRI) Triton SS-OCT (Topcon Corporation, Tokyo, Japan). To evaluate SCP, DCP and CC VD, the 3×3 mm OCTA protocol was performed with IMAGEnet 6 Version software 1.22.1.14101^®^ 2014 (Topcon Corporation). The FAZ from the SCP and DCP images (Figure 1) was obtained for manual delimitation.

### 2.3. FAZ Segmentation

**Figure 2:**
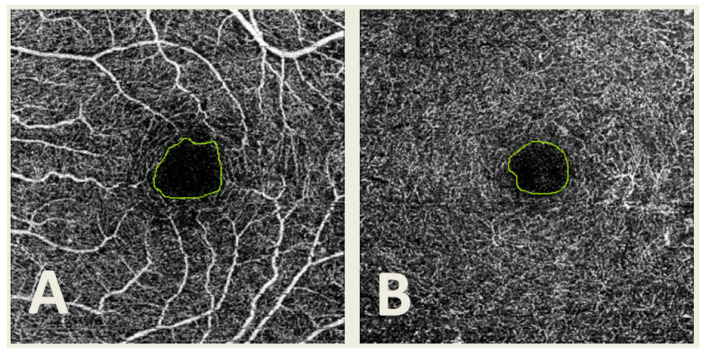
Optical coherence tomography angiography (OCTA) image from the superficial capillary plexus (**A**) and deep capillary plexus (**B**) with the foveal avascular zone (FAZ) manually delimited.

The SCP and DCP images were obtained using IMAGEnet 6 Version software 1.22.1.14101^®^ 2014, and then they were exported in TIFF format. Then, manual delimitation of the FAZ was performed by four different observers (G.F.-E., F.J.S.-R., C.R.-T. and E.O.-H.) using the same computer and Microsoft Paint software version 22H2 for Windows 10 (Microsoft Corporation, Redmond, WA, USA) in both SCP and DCP (Figure 2). A total of 3222 segmentations were obtained.

**Figure 3:**
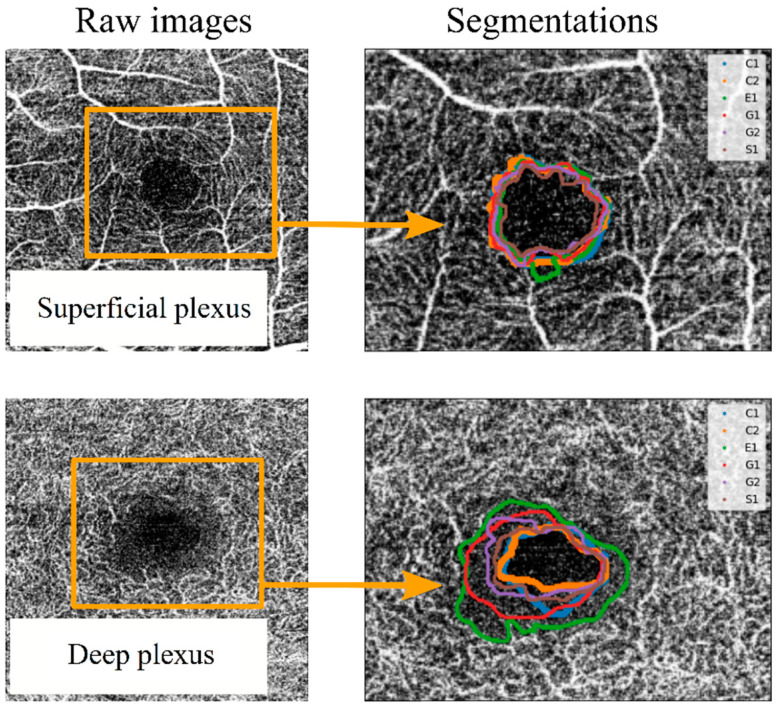
Example of superposition of the different observers’ segmentations for a superficial capillary plexus (SCP) OCTA image (**top**) and for a deep capillary plexus (DCP) OCTA image (**bottom**). To differentiate the perimeter of the FAZ between observers, different colour lines were used, labelled C1, C2, E1, G1, G2 and S1. In this labelling, each letter corresponds to an observer, and the number corresponds to different segmentation series.

As a primary approach, the FAZ was defined as “the region within the fovea without blood vessels”. Each observer segmented the images set one or two times (depending on the observer) without any instructions or external influences. Each segmentation was labelled with a letter (corresponding to the four observers) and a number (indicating the number of measurement series for each observer). Thus, the measurements labelled “G1” and “G2” correspond to segmentations made by the same observer in different measurement series. Once the segmentation process was completed, the spatial coordinates of the perimeter line over the image for each segmentation were extracted. These coordinates allowed the comparison between segmentation series. For example, in Figure 3, the FAZ perimeter lines were collected for two images (corresponding to the SCP and the DCP of the same eye of a patient affected by DM1), and they were drawn superimposed on the corresponding image. Variability between different segmentations was observed, as shown in Figure 3. Some observers tended to adjust the FAZ perimeter to the enclosing blood vessel, leading to larger areas and more sinuous lines. In the SCP, the variability increased when an enclosing vessel ended in a bifurcation. In the DCP, differences between different observers were higher, as the vessels correspond to the most internal retinal vascularisation (with anastomosis to the SCP), making it more difficult to subjectively delimit the FAZ.

The OCTA-acquired image results from a volumetric layer of a certain thickness. For this reason, the boundary between the nonvascular and vascularised zones appears as a transition zone rather than an abrupt change. In this transition zone, it was still possible to find blood vessels, at least in deeper layers. Furthermore, the area surrounding the vessels was irrigated, for which reason the FAZ was delimited by slightly entering its limits from the clearly marked vascularisation. To improve agreement among observers, a new delimitation of the FAZ was proposed as “the inner boundary of the transition zone between the avascular area and the irrigated area of the fovea”.

Then, observers were asked again to perform segmentations on the same set of images after a reasonable period of time. Thus, a new set of boundary coordinates was obtained for statistical analysis to establish comparisons between observers, criteria and groups of patients.

**Figure 4:**
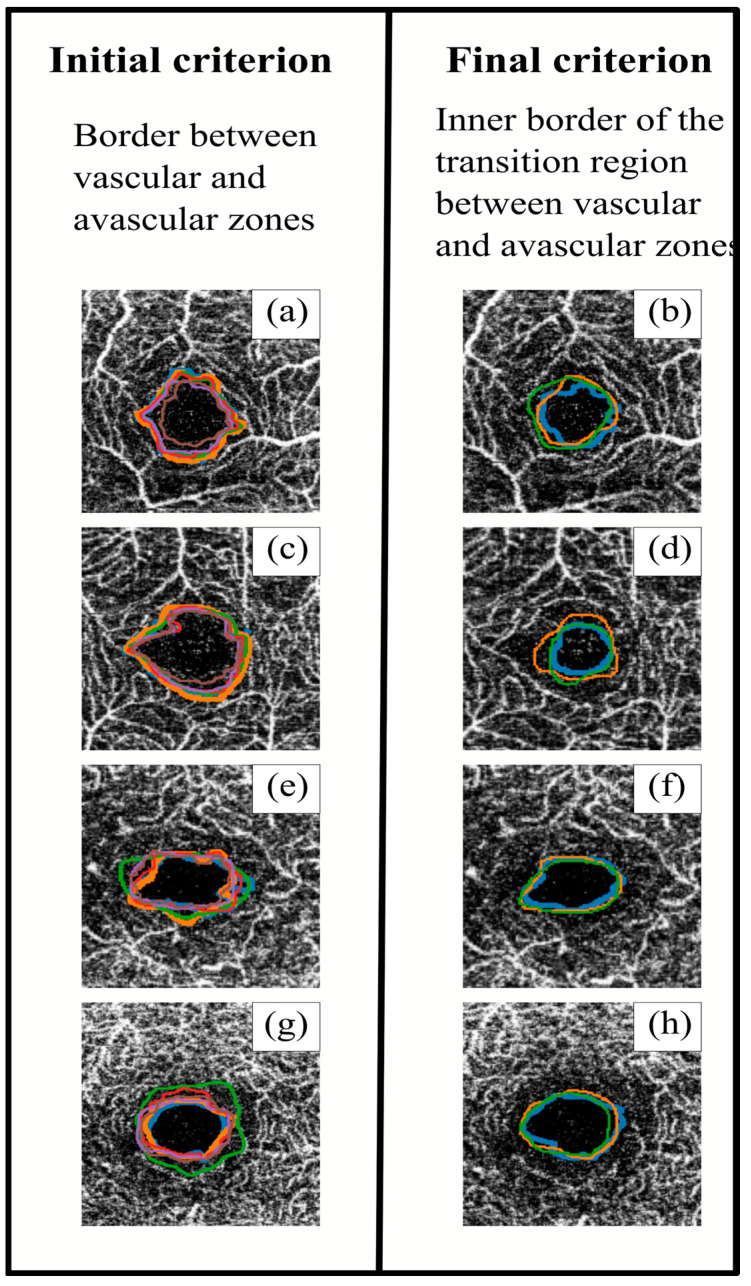
Superposition of the different observers’ segmentations using the initial (**left**) and final (**right**) criteria. The images in the same row correspond to the same sample. Images (**a**–**d**) correspond to superficial capillary plexus (SCP), and images (**e**–**h**) correspond to deep capillary plexus (DCP). Each colour line corresponds to a different observer.

Figure 4 shows an example of segmentations obtained with both initial and final criteria. After applying the final criterion, the segmented perimeters tended to be more homogeneous (both between different observers and between the same observers), with slightly smaller areas. At the same time, the perimeter lines with the initial criterion tended to follow the blood vessels, resulting in spikier shapes, while the final criterion produced smoother shapes. The definition of a common segmentation criterion would achieve better FAZ contouring. This point is really important, since in order to continue advancement in retinal research, it is very important to have a good base of segmented images in which the variability between measurements is reduced.

An objective comparison was made between the two methods to define the FAZ. Two diagnostic parameters were used to compare the segmentations: the area and the acircularity. The first was the FAZ area, for which incorrect segmentation was a serious impediment to its use as a biomarker, as the literature attests [15,16].

The second parameter was the acircularity, related to the shape of the FAZ. In general terms, the acircularity of a curve is defined as the first moment of its radial coordinates. Thus, it provides information about the similarity between a closed curve (of segmented perimeters) and a perfect circumference with a mean radius. Given a segmented FAZ, the coordinates of the perimeter line were used to obtain the centre of the curve, the mean distance between the centre and the radial coordinate of each point in the curve (i.e., the mean radius of the curve), and the difference between the distances and the mean radius. Then, acircularity was computed as follows:

(1) $$Acircularity=\frac{\sigma \left(R\right)}{\langle R\rangle },$$

where $\langle R\rangle$ is the mean distance between the centre and the N points in the curve (the mean radial coordinate),

(2) $$\langle R\rangle =\frac{1}{N}\sum_{i=1}^{N}R_{i},$$

and σ(R) is the standard deviation of the radial coordinates,

(3) $$\sigma \left(R\right)=\sqrt{\frac{1}{N}{\left(R_{i}-\langle R\rangle \right)}^{2}}.$$

If the contour were a perfect circle, the standard deviation of the distance of the contour to the centre would be zero, as the contour would be at a constant distance (radius) from the centre.

### 2.4. Statistical Analysis

Once we have a set of segmented images by all the observers (using both criteria), the statistical analysis was automatised using a code written in Python (Python Software Foundation, Python Language Reference, version 3.6) to obtain the area and acircularity values for each segmentation. We provide an example code in the Supplementary Materials (Figure S1 and Example Code S2), and we give here a deeper explanation of the process.

Firstly, in order to compute the statistics for each contour, the coordinates of the FAZ contour must be extracted from the image. To get the coordinates correctly sorted, we transform the Cartesian locations of the points in the contour into polar coordinates and we sort them according to their azimuth. Then, we switch back to Cartesian coordinates, this time correctly sorted.

**Figure 5:**
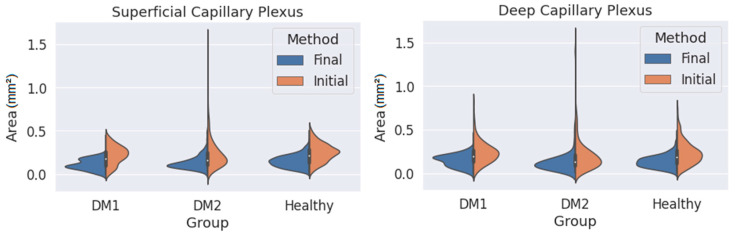
Measured area values obtained for the three groups (DM1, DM2 and healthy subjects) using both criteria (orange: initial criterion; blue: final criterion) and for the superficial capillary plexus (SCP) (**left**) and deep capillary plexus (DCP) (**right**).

Next, the coordinates are organised in two same-length arrays, containing the x and y coordinates of each point, their length being the total number of points that form the contour. Then, the internal area of this contour (which forms a polygon) can be calculated using the shoelace method (also known as the Gauss method). Thus, if we have n pairs of coordinates, we apply,

(4) $$Area=\frac{1}{2}\sum_{i=1}^{n-1}\left(y_{i}+y_{i+1}\right)\left(x_{i}-x_{i+1}\right)+\left(y_{n}+y_{1}\right)\left(x_{n}-x_{1}\right)$$

where each sub-index indicates the coordinates of the i-th point on the contour. This area is calculated in square pixels, so we transform it to square millimetres using the pixel size provided by the Triton OCT manufacturer. Using this method, it is possible to obtain the area of any closed contour conformed by ‘*n*’ points. This value is computed for every contour in the dataset and organised depending on the patient type (DM1, DM2 or healthy) and on the observer and the method (initial or final), and then the area distribution is plotted as a violinplot (Figure 5) showing the statistical occurrence of the values. We also provide the main statistical parameters (mean area and standard deviation) in Table 1.

A similar process is used to obtain acircularity values. In this case, once we have a sorted set of coordinates for an FAZ contour, the geometrical centre of this contour is obtained by computing the average of the coordinates of all the points in the contour, and we take the coordinates at this central point as the origin. Then, once the contour is centred at the origin, we transform again the Cartesian coordinates to polar coordinates. Now, the acircularity of each contour can be easily calculated from the radial coordinate array, using Equation (1) and R being the radial coordinate array. 

**Figure 6:**
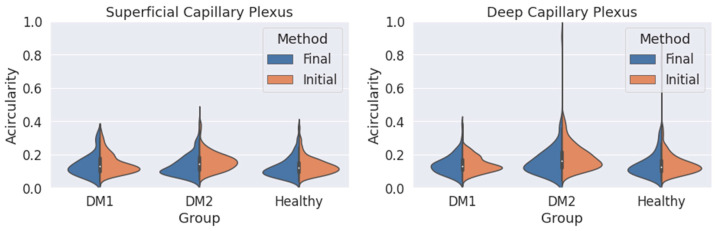
Measured circularity values obtained for the three groups (DM1, DM2 and healthy subjects) using both criteria (orange: initial criterion; blue: final criterion) for the superficial capillary plexus (SCP) (**left**) and deep capillary plexus (DCP) (**right**).

We obtain an acircularity value for each contour. Repeating this process for all the segmentations, we extract the statistical values applying Equations (2) and (3), and we show the results in Figure 6 and Table 2.

## 3. Results

The mean age was 41.70 ± 11.49 years (range 21–66 years) for the 40 DM1 patients, 64.06 ± 11.98 years (range 42–86 years) for the 54 DM2 patients and 60.79 ± 8.62 years (range 42–83 years) for the 73 healthy patients (the control group). The gender distribution was 39.7%, 50% and 20.4% females and 60.3%, 50% and 79.6% males in the control, DM1 and DM2 groups, respectively. DM1 and DM2 patients were endocrinologically well controlled, with a mean HbA1c of 7.68 ± 0.98% in the DM1 group and 7.58 ± 1.29% in the DM2 group; the mean time since diabetes diagnosis was 26.7 ± 7.84 years (range 11–43 years) for the DM1 group and 2.50 ± 2.88 years (range 0–11 years) for the DM2 group.

The first analysis was related to the segmented FAZ area. Figure 5 shows the distribution of the area data obtained for the three groups (DM1, DM2 and the healthy subjects) in the SCP and DCP. The values obtained when using the initial and final criteria are also shown (in orange and blue, respectively).

As seen in Figure 5, the final criterion produces smaller areas than the initial one in both plexuses for the three groups. At the same time, the variability of the results (i.e., the width of the distribution curves) was lower with the final criterion. This was particularly noticeable for the DM2 group, with greater changes in the FAZ, where the FAZ was difficult to delimit with the initial criteria. The reduction in variability was appreciable even in the group of healthy patients. The long tails of the distribution curves correspond to isolated values far from the average. If we focus only on the final criterion, the variability in the areas for the SCP was slightly smaller than for the DCP, indicating that the transition zone was clearer in the SCP.

Table 1 presents some numerical values extracted from Figure 5, showing a lower mean area and SD with the final method. The average areas were also noticeably smaller when the final method was applied. Assuming that the values obtained with the final criterion were more reliable, in the case of SCP, there was no increase in area in the DM1 and DM2 groups compared with the control group (in fact, for the DM1 group, the average area was reduced). The same can be said for the DCP. These results seem to contradict the findings of other studies that found an enlargement of the FAZ in DM patients compared with healthy subjects [17,18,19]. In any case, our results with the initial criterion (which is typically applied) also point in the same direction. In any case, the reduction in standard deviation is notable, which means that our segmentations are more consistent. This represents a great advance, since we now have a correctly segmented images database.

We also studied the acircularity of the FAZ (Figure 6). Regarding the SCP, the values and the standard deviations (SD) were similar with both methods. The largest differences between both methods were found in the DM2 group, with a significant reduction in the mean acircularity value. In DCP, the extreme values obtained with the initial method were reduced with the final method. Again, the mean values were very similar for the three groups and both methods. The mean values and the standard deviation for the acircularity measurements are shown in Table 2. The acircularity values were slightly reduced with the use of the final criterion, although not as significantly as in the area value.

**Figure 7:**
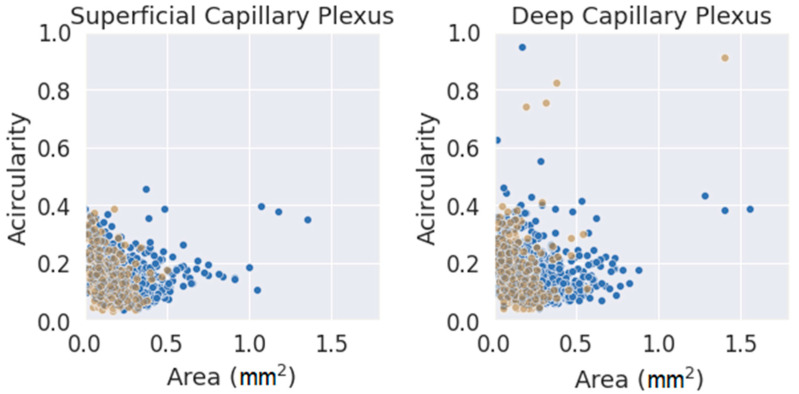
Area vs. acircularity for each plexus. In blue, the measurements performed with the provisional method. Blue dots were obtained with the initial method, and brown dots were obtained with the definitive method.

Finally, and as a final comment, a comparison between the two parameters used (FAZ area and acircularity) was performed, as shown in Figure 7. The values were classified by segmentation criterion and by layer. The data seemed to fit a clear trend in both layers, showing slightly higher values of acircularity for smaller areas. The number of extreme values (far away from the average of both parameters) was much higher for the initial method (blue circles) and for DCP. Note that an acircularity value above 0.5 denotes a very irregular FAZ (this may be due to poor segmentation or an FAZ that was effectively far from a pure circular shape). Discarding these extreme values, the measurements obtained with the criterion presented in this work were more clustered in a more compact pattern, adjusting better to the trend. Therefore, the new criteria for FAZ segmentation were more reliable and produced more consistent results. This may be because it was based on a segmentation criterion that was closer to reality; at the same time, it was easier to understand correctly by any observer. This fact is very important as a starting point for future research.

## 4. Discussion

OCTA has been implemented as a routine technique in clinical practice. However, the interpretation of its measurements is not entirely free of risks, as a manual method is usually necessary to delineate the retinal areas of interest. In the best case, software tools are available to perform this segmentation, which in the end are also dependent on a correct definition of the parameters.

DRI-Triton SS-OCT and the 3×3 mm OCTA protocol allowed us to study the FAZ in both retinal plexuses, the SCP and DCP. The FAZ can present different changes, such as morphology or capillary perfusion, in DM and DR, as is the case for our patients in the DM2 group [20,21,22].

In our research, we studied the variability of the manual delimitations of the FAZ measured by OCTA in DM2 patients with moderate NPDR without diabetic macular edema (DME), DM1 patients without DR and healthy subjects. The variability of the results was quite elevated when the standard delimitation of FAZ was used. We understood that the cause of the discrepancies was the lack of accuracy in the definition.

For this reason, a new criterion for FAZ segmentation was proposed that does not exclude the initial definition but rather complements it. The images obtained with OCTA reflect the vascularity in a volumetric layer. Near the FAZ, where the superficial vessels begin to disappear, some vascularity from more internal vessels was still present. Therefore, an irrigated zone, a transition zone and an inner zone, which was the FAZ itself, were differentiated. Using this novel criterion, which is more in line with reality and at the same time more precise, more homogeneous results were obtained, with less variability in the segmentations (both intraobserver and interobserver).

Given the improvement in the segmentations, the measurements obtained with the final method were more reliable than those obtained with the initial criterion; therefore, the values collected in this work may be of interest to the clinical community. Some commentaries can be drawn from our statistical analysis. Independent of the observer or the method used, an increase in the acircularity value was observed when the area increased. One possible explanation could be that for a large FAZ, the contour became more irregular. Hence, while those irregularities increased the total area, the contour deviated from a perfect circle; thus, the acircularity value increased. The same was applied in the opposite case, as smaller FAZ contours tended to be segmented with smoother lines resembling a circle, decreasing acircularity.

Moreover, a variation between segmentation methods was also observed. From Figure 5, the initial method produced much less homogeneous results in the measurement of the FAZ area than those obtained with the definitive method. As the area was a crucial parameter in the control of DR progression, the use of our new criterion for FAZ segmentation could help in clinical practice.

Although the FAZ usually appears as a simple dark area in the centre of the image in healthy subjects, depending on the criteria used by each observer for the manual segmentation of the FAZ, very different results can be obtained. This is even more difficult in the eyes of diabetic patients as they may present more irregular FAZ or anatomical alterations and retinal vascularisation abnormalities [19,23]. In addition, in the advanced stages of DR, the FAZ is enlarged, and the MA around it develops in cystoid macular edema eyes, which makes its segmentation even more difficult [24]. An enlarged FAZ and reduced vascular density have even been shown to be predictors of poor visual outcomes after anti-vascular endothelial growth factor (VEGF) treatment for DME [25,26,27].

Currently, the manual delimitation of the FAZ is still considered the gold standard in clinical practice. Authors such as Mirshahi et al. [28] studied the possible delimitation of the FAZ automatically and through deep learning, obtaining good results in healthy subjects but poor results for the delimitation of the FAZ in diabetic patients. The presence of various artefacts, especially in diabetic eyes, may interfere with the accuracy of the available automated method [28].

Although the excellent reproducibility of the FAZ in healthy subjects has been reported, intraobserver and interobserver variability in diseased eyes needs to be assessed in future studies [14]. Authors such as Guo et al. proposed and successfully verified an automatic FAZ segmentation and quantification method based on deep learning in a group of myopic patients [29]. Other authors, such as Díaz et al. [30], also studied the automated segmentation of the FAZ in healthy and diabetic patients and their comparison with manual segmentation, obtaining poor results in the FAZ that were irregular and did not follow the acircularity, especially in the 3 × 3 $mm^{2}$ OCTA protocol. Although many studies are available regarding different image processing techniques for automatic segmentation of the FAZ in various retinal imaging modalities [30,31,32,33,34,35,36], studies focusing on automatic FAZ segmentation in OCTA were usually conducted on healthy subjects. In addition, a few studies assessing the accuracy of FAZ delineation in OCTA images of diabetic eyes have obtained poor results due to the high incidence of signal noise and artefacts in OCTA imaging of diabetic patients [7,8,9,10,11,12,13,14,15,16,17,18,19,20,21,22,23,24,25,26,27,28,29,30,31,32,33,34,35,36,37,38,39]. However, some authors such as Liu et al. proposed the application of the watershed algorithm in FAZ segmentation for analysing and diagnosing eye diseases [40].

The automatic segmentation of the FAZ could be another possible line of study in the future that could be applied in daily clinical practice, but for the moment the most commonly used method is manual segmentation of the FAZ, which varies according to the criteria of delimitation that the observer uses.

Another point to take into account is the type of OCT device which has been used, since authors such as Corvi et al. found that comparison between instruments is almost impossible and that the measurement set of the various instruments are not interchangeable with respect to vascular density and FAZ for the SCP and DCP [41].

As a final remark, it is necessary to continue in this line of work as the results obtained in our study are quite reliable. With the segmentations obtained, which show less variability and therefore seem more accurate, it is possible to obtain much more information than that presented in this article. In this way, we have a set of images with reliable segmentations, which we can continue to expand in the future. This represents a solid starting point for further research, both from a diagnostic point of view (since reliable statistical data can be extracted) and from the point of view of segmentation automation (since we have a set of correct segmentations to compare with). We are sure that the method presented can be used by the clinical community in future works.

## 5. Conclusions

In conclusion, the implementation of a new criterion for FAZ segmentation was more reliable and accurate and produced more consistent results with less variability among observers. It was based on a delimitation that is closer to reality and easier to understand correctly by any observer. Therefore, we now have a very reliable set of segmentations with which we can continue our research. Thus, for example, we can use these measurements to extract reliable statistics to define new biomarkers for use in disease diagnosis, but we can also use these segmentations with low variability to improve the accuracy of new automatic segmentation tools.

## Figures and Tables

**Table 1:** Average (mean) and standard deviation (SD) of the foveal avascular zone (FAZ) areas (in $mm^{2}$ obtained for the different groups and layers using the initial and final criteria.

Group	Method	SCP		DCP	
		$\mathrm{Mean}\;(mm^{2})$	$\mathrm{SD}\;(mm^{2})$	$\mathrm{Mean}\;(mm^{2})$	$\mathrm{SD}\;(mm^{2})$
DM1	Initial	0.22	0.11	0.24	0.13
	Final	0.11	0.06	0.15	0.07
DM2	Initial	0.25	0.19	0.21	0.19
	Final	0.14	0.08	0.13	0.13
Healthy	Initial	0.25	0.11	0.24	0.14
	Final	0.15	0.07	0.14	0.07

**Table 2:** Average (mean) and standard deviation (SD) of the FAZ acircularity values obtained for the different groups and layers using the initial and final criteria.

Group	Method	SCP		DCP	
		Mean	SD	Mean	SD
DM1	Initial	0.15	0.06	0.14	0.05
	Final	0.14	0.07	0.14	0.06
DM2	Initial	0.16	0.06	0.19	0.09
	Final	0.14	0.06	0.16	0.09
Healthy	Initial	0.13	0.05	0.14	0.07
	Final	0.13	0.06	0.14	0.09

## Data Availability

The data presented in this study are available within the article.

## Supplementary Materials

The following supporting information can be downloaded at: https://www.mdpi.com/article/10.3390/jpm13050822/s1, Figure S1. Example manual segmentation. Code S2. Example calculations.
