# Supplementary figures and images for: A Reliable Criterion for the Correct Delimitation of the Foveal Avascular Zone in Diabetic Patients
Source: J Pers Med. 2023 May 12;13(5):822. doi: 10.3390/jpm13050822 (PMC10223277; doi:10.3390/jpm13050822)

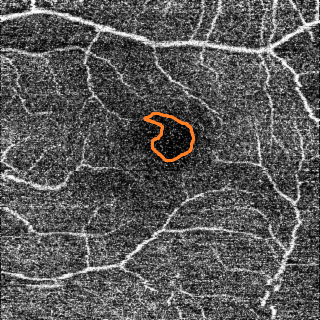

Supplement: Supplementary file 1 [file jpm-13-00822-s001.zip › Figure S1_example_manual_segmentation.tif]
